# Analysis of 9 Cases of Takotsubo Syndrome and an Analysis of the Clinical Characteristics of Takotsubo Syndrome From a Chinese Population

**DOI:** 10.3389/fcvm.2021.732193

**Published:** 2021-10-26

**Authors:** Ju Yan, Mahesutihan Madina, Changjiang Deng, Qianru Yuan, Shixiong Cao, Xiang Xie, Yitong Ma

**Affiliations:** ^1^The First Affiliated Hospital of Xinjiang Medical University, Ürümqi, China; ^2^Department of Cardiology, The First Affiliated Hospital of Xinjiang Medical University, Ürümqi, China

**Keywords:** Takotsubo syndrome, stress cardiomyopathy, broken heart syndrome, case analysis, clinical characteristics

## Abstract

**Objective:** To summarize the clinical features, hematology and imaging features of Takotsubo syndrome.

**Methods:** The hospitalization data of Takotsubo syndrome patients in the First Affiliated Hospital of Xinjiang Medical University from January 2015 to December 2020 were collected, and their clinical characteristics were summarized. Patient outcomes were clarified through follow-up visits, and relevant objective indicators were statistically analyzed before and after admission. The characteristics of TTS incidence in Chinese population were summarized by searching three (Wanfang, CNKI, China's VIP database) major databases in China (PRISMA).

**Results:** A total of 9 patients were enrolled, including 6 females (66.7%). The mean age of onset was 46.4 years old, the median time from onset to treatment was 1 day. The main symptom of 8 cases (88.9%) was chest pain, 1 case had a main symptom of syncope, and 7 cases (77.8%) had mood fluctuations or mental stimulation as the main symptom of the disease. Paired *T*-tests were conducted on routine blood, biochemical, coagulation, myocardial markers, inflammatory indicators and objective indicators of ECG before and after admission. The study found that the counts of white blood cells and neutrophils were statistically significant (*P* < 0.05). Prolongation of the QT interval was observed in all 9 patients. After a mean follow-up of 24 ± 28 months, no adverse cardiovascular events or recurrence occurred.

**Conclusion:** Takotsubo syndrome is a group of clinical syndromes with emotional or somatic stimulation and chest pain as the main symptoms, partly accompanied by an increase in white blood cells, neutrophilic granulocyte count, creatine kinase, and troponin and is characterized by a prolonged QT interval and no obvious coronary stenosis. The prognosis is generally good, with few serious complications.

Takotsubo syndrome (TTS) is also known as stress cardiomyopathy, apex spherical syndrome, octopus pot cardiomyopathy, broken heart syndrome, stress-induced cardiomyopathy, and Holy Bottle syndrome. Due to the continuous development of society, people's pressure is increasing, which is easy to induce Takotsubo syndrome. The clinical symptoms and electrocardiographic manifestations of this syndrome are similar to those of acute coronary syndrome. The main characteristic of this syndrome is regional abnormal left ventricular wall motion accompanied by abnormal left ventricular dilation during the systolic period. After the removal of the related inducements, the cardiac function of the patient returns to normal, and the prognosis is generally good. By retrospectively collecting the baseline data, clinical data and follow-up of patients with Takotsubo syndrome, we summarized the relevant clinical features of Takotsubo syndrome to facilitate more medical personnel to understand Takotsubo syndrome and to provide clinical guidance, since the current diagnostic method is mainly by a diagnosis by exclusion.

## Case Data and Methods

### Inclusion and Exclusion

Inclusion criteria were mainly based on the criteria for Takotsubo syndrome published by the Mayo Clinic; the main contents are as follows: (1) transient hypokinesia or abnormal movement in the middle segment of the left ventricle with or without apical involvement. Abnormal regional wall movement exceeds the distribution of single epicardial vessels. Triggers of stress are often present, but not always. (2) No angiographic evidence of obstructive coronary artery disease or acute plaque rupture. (3) New ECG abnormalities (ST-segment elevation and/or T-wave inversion) or mild myocardial troponin elevation (1). The exclusion criteria were as follows: (1) patients with a definite diagnosis of CHD by coronary angiography; (2) patients with abnormal left ventricular wall motion consistent with a single coronary artery region; (3) pheochromocytoma and myocarditis; and (4) patients with poor compliance. If the inclusion of a patient was controversial, the patient was reviewed by all members of the TTS team to reach a consensus.

### Observation Indicators

Age of onset, sex, ethnicity, admission time, admission method, initial clinical manifestations (chief complaint, main symptoms, inducement, location, size, nature, duration, aggravating or mitigating factors, concomitant symptoms), previous medical history, admission vital signs, height and weight of all patients were recorded. Serological examination results (routine blood, biochemical, myocardial enzyme, troponin, coagulation set, CRP, PLT, IL-6, etc.), auxiliary examination (ECG, cardiac ultrasound, coronary angiography, myocardial nuclide scan, cervical vascular ultrasound, etc.,) and follow-up to observe whether the patient has recurrence or aggravation were also recorded.

### Research Methods

This study was a retrospective, non-case-control follow-up study. The clinical data of Takotsubo syndrome patients in the First Affiliated Hospital of Xinjiang Medical University from January 2015 to December 2020 were collected, and the descriptive statistical analysis was conducted using Excel and SPSS 21.0. For measurement data, a normal test was carried out first, and the means plus standard deviations was used to describe those data following a normal distribution. Paired *T*-tests were used for the same patient from admission to discharge. The factors that did not follow a normal distribution are represented by the median (P25, P75), and the comparison between the two groups was performed by the Mann-Whitney test. For the enumeration data, we described the composition of the index by calculating its constituent ratio (%). A mean follow-up of 24 ± 28 months was followed for recurrence or exacerbation.

### The Clinical Characteristics of Takotsubo Syndrome in Chinese Population Were Searched Through Three Major Databases in Chinese

**Figure 1:**
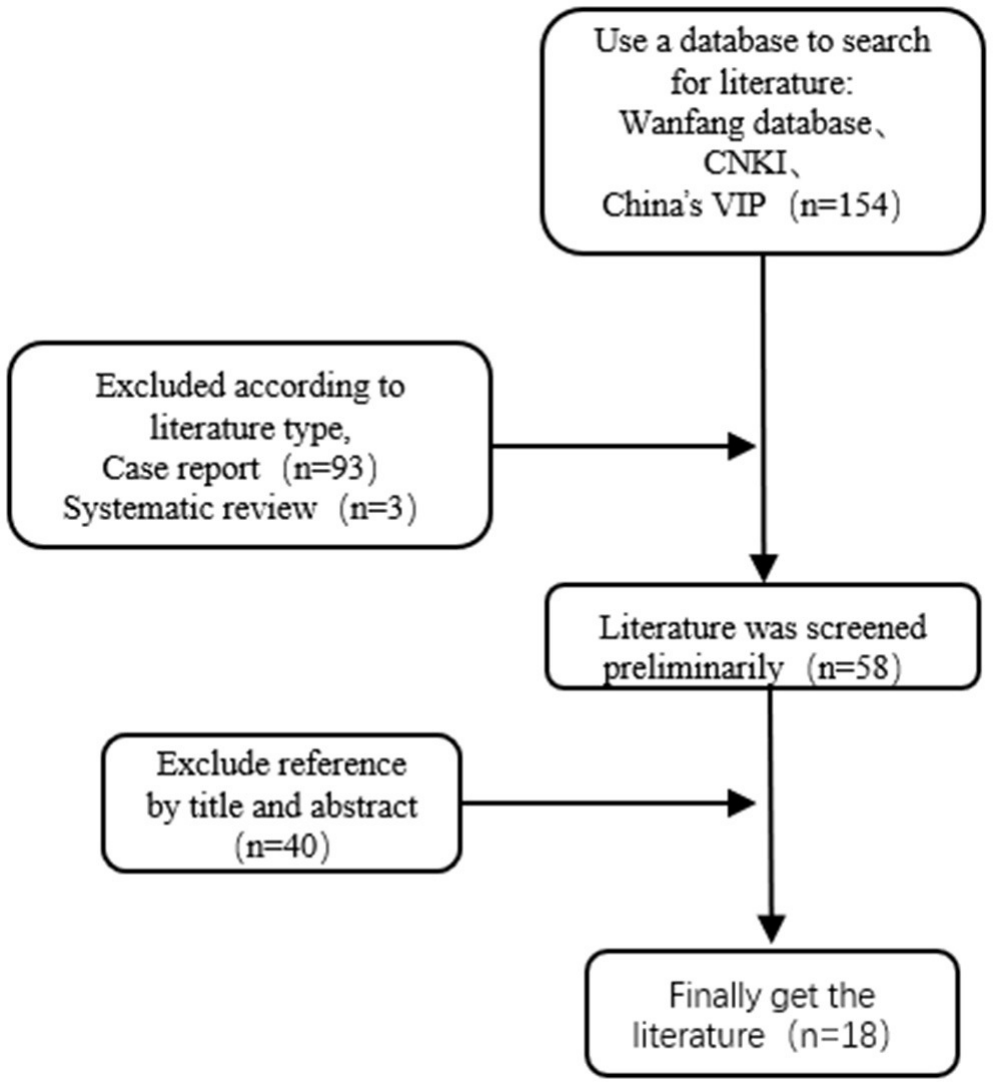
Inclusion of literature process.

By searching three major databases: Wanfang, CNKI and China's VIP database. The search terms were “stress cardiomyopathy” or “Takotsubo syndrome” or “broken heart syndrome” or “apical spherical syndrome” or “octopus pot cardiomyopathy” or “stress-induced cardiomyopathy” or “Holy Bottle syndrome.” Chinese literatures published before April 31, 2021 were selected for reading, and their references were supplemented for reading. Inclusion criteria were consistent with the diagnostic criteria published by the Mayo Clinic. Exclusion criteria patients also had other diseases, and its influence on stress cardiomyopathy could not be excluded; Exclusion case report; Exclusion of cases ≤5; Exclude by title and abstract. From 8 dimensions of “gender, age, risk factors, symptoms, ECG, UCG, LVG, and follow-up” through literature retrieval, the clinical characteristics in each dimension were statistically summarized. A total of 154 literatures were initially examined. According to the inclusion and exclusion criteria, 18 literatures were finally included, including 264 cases, as shown in Figure 1. The literature retrieval process was shown in Figure 1, and the clinical characteristics were shown in Table 1.

**Table 1:** Onset characteristics of Takotsubo syndrome in some Chinese population.

**References**	**Cases**	**Gender**	**Age**	**Incentive**	**Symptoms**	**ECG**	**UCG**	**LVG**	**Follow up**
Tan and Luo (2)	5	F (100%)	57–76	ES^*^:3 cases	Chest pain	ST-SE and T-WI	EF: 19–34%, the apical movement disappeared in 2 cases and decreased to flat in 3 cases	Left ventricular angiography showed bulbous apex dilatation with significantly reduced or absent movement	7–33 m, no recurrence occurred in 5 patients
Mo (3)	27	F (66.7)	21–74	ES: 10 cases, PS^#^: 17 cases	Chest pain, fainting, difficulty breathing	ST-SE^a^: 7 cases, ST-SD^b^: 20 cases, T-WI^c^: 7 cases, QTC-IP^d^: 7 cases	EF decreased significantly, and the apical and mid-segment motion abnormalities of the left ventricle were found in 7 cases	5 cases of left ventricular angiography showed that the apex of the left ventricle was bulbous	21 d, no recurrence occurred in 27 patients
Sun and Xuan (4)	6	F (83.3%)	48–72	ES: 4 cases, PS: 2 cases	Chest pain, fainting shortness of breath, difficulty breathing	ST-SE and T-WI	EF: 20–35%, the apical movement disappeared in 3 cases, and the movement significantly weakened to flat in 3 cases	No coronary angiography was done	7–11 d, LVEF and apical wall motion returned to normal
Gao et al. (5)	9	F (77.8%)	42–67	ES: 3 cases, FS: 6 cases	Chest pain, difficulty breathing, fainting	ST-SE: 2 cases, ST-SD: 6 cases, abnormal Q wave in 1 case	EF: 30–45%. Absence of apical movement and reduced segmental movement of ventricular wall	Coronary angiography showed no obvious abnormality	7–14 d, LVEF and ventricular wall movement were restored, and 2 cases had slight decrease in the apex
Liu et al. (6)	10	F (90%)	49–83	ES: 8 cases, FS: 2 cases	Chest pain, fainting	ST-SE: 10 patients, QTC-IP was prolonged	EF: 46.67 ± 1.41%. Segmental ventricular wall movement was abnormal at the apex	9 cases of left ventricular angiography showed bulbous apex dilatation	1.65 ± 1.78 y, no recurrence occurred in 10 patients
Yang et al. (7)	20	F (85%)	53–74	Psychological stress	Chest pain, chest tightness	ST-SE: 20 cases, T-WI: 13 cases, QTc-IP: 5 cases	EF: 30–41%, The ventricular wall motion at the apical part of the heart is weakened	Left ventricular angiography showed spherical apex dilatation and segmental ventricular wall motion abnormality	1–3 y, 20 patients were followed up for 1 year without recurrence, and 8 patients were followed up for 3 years without recurrence
Wang (8)	14	F (71.4%)	41–79	Subarachnoid hemorrhage	Chest tightness, Chest pain, dyspnea, fainting	ST-SE: 7 cases, ST-SD: 3 cases, T-WI: 11 cases, QTc-IP: 5 cases	EF: 36.07 ± 6.15%, left ventricular apical and middle systolic dysfunction	Left ventricular angiography was not performed	1 m, no recurrence occurred in 14 patients
Yi and Ye (9)	8	F (75%)	30–71	ES: 3 cases, FS: 5 cases	Chest pain, fainting, difficulty breathing	ST-SE: 2 cases, ST-SD: 6 cases, T-WI: 4 cases	EF: 30–40%, 4 cases of left ventricular wall motion abnormality, 3 cases of apical balloonoid change	Left ventricular angiography indicated Lvef 40%−50%, weakened ventricular wall and apex movement	21 d, LVEF and ventricular wall motion returned to normal
Gan and Zhang (10)	10	F (90%)	50–81	ES: 8 cases, FS: 2 cases	Chest pain, chest tightness, dyspnea and fainting	ST-SE: 8 cases, ST-SD: 2 cases, T-WI: 8 cases, QTc-IP: 5 cases	EF: 25–41%, disappeared apical motion in 5 cases, weakened motion in 5 cases, balloon-like change in 3 cases, and contradictory motion in 7 cases	Left ventricular angiography showed that the apex of the left ventricle was bulbous and the apex motion was weakened or disappeared	8–12 m, no recurrence occurred in 10 patients
Le (11)	15	F (80%)	38–72	ES: 6 cases, FS: 9 cases	Chest pain, fainting, difficulty breathing	ST-SE: 7 cases, T-WI: 8 cases	The apical and middle parts of the left ventricle showed balloon-like changes, and the motion was obviously weakened or disappeared or showed contradictory motion	Coronary angiography was performed in 6 cases and CTA was performed in 9 cases, and no significant coronary artery stenosis was observed	N
Hou (12)	9	F (77.8%)	68.44	ES: 4 cases, FS: 2 cases	Chest tightness, chest pain, transient confusion	ST-SE: 4 cases, ST-SD: 2 cases, T-WI with QTc-IP: 1 cases	There were 4 cases of segmental ventricular wall motion decrease, and the remaining 5 cases showed left ventricular motion decrease or left ventricular systolic (diastolic) function decrease	Left ventricular angiography showed spherical apex dilatation in 1 case, disappearance of apex activity in 1 case, and weakening of anterior wall and apex activity in 1 case	3 m−2 y, 1 case died, 4 cases had no recurrence, and 1 case was lost to follow-up
Hu et al. (13)	10	F (80%)	28–72	ES: 5 cases, FS: 5 cases	Chest pain, difficulty breathing	ST-SE: 7 cases, ST-SD: 2 cases, T-WI: 1 cases	EF: 15–39%. Absence of apical movement or reverse movement	Four cases of left ventricular angiography showed that the apex of the heart was spherical dilatation, and the movement was obviously weakened or disappeared	7–10 d, LVEF and ventricular wall movement returned to normal
Jiang and Ning (14)	20	F (80%)	47–63	ES: 10 cases, PS: 10 cases	Chest pain, difficulty breathing	ST-SE: 14 cases	EF: 28%, absence of apical movement	Left ventricular angiography showed bulbous apex dilatation	7–1 0d, LVEF and ventricular wall movement returned to normal
Li (15)	12	F (83.3%)	23–67	ES: 8 cases, FS: 4 cases	Chest pain, fainting, chest tightness, palpitations, dyspnea	ST-SE with T-WI: 8 cases, ST-SD: 2 cases	EF: 30–45%, weakened ventricular wall motion in 7 cases, contradictory motion in 3 cases, and bulbous apex dilatation in 2 cases	Left ventricular angiography showed bulbous apex dilatation	1 m, LVEF and ventricular wall movement returned to normal
Han et al. (16)	16	F (87.5%)	N	ES: 6 cases, FS: 10 cases	Chest pain, retrosternal pain, chest tightness	ST-SE: 8 cases, T-WI: 8 cases	The apical and middle parts of the left ventricle showed balloon-like changes, and the wall motion was obviously weakened or disappeared	Coronary angiography showed no significant coronary stenosis	2–4 w, ventricular wall movement returned to normal
Lu et al. (17)	8	F (75%)	40–76	ES: 5 cases, FS: 3 cases	Chest pain, weakness, palpitations, difficulty breathing	ST-SE: 6 cases, T-WI: 2 cases, QTc-IP: 5 cases	EF: 27–40%, 5 cases showed bulbous dilatation at the apex or middle of the heart and uncoordinated movement of the ventricular wall	Coronary angiography was normal in 6 cases, and single coronary artery stenosis in 2 cases was 30 and 50%, respectively	1 m, LVEF and ventricular wall movement returned to normal
Feng et al. (18)	52	F (84.6%)	30–82	ES: 26 cases, FS: 5 cases, ES and FS: 5 cases	Chest pain, dyspnea, chest tightness, palpitations, fainting	ST-SE: 48 cases, T-WI: 22 cases, QTc-IP: 5 cases	EF: 21–54%, 38 cases showed spherical expansion of the apex of the left ventricle, weakened or disappeared movement	30 cases of left ventricular angiography showed that the apex of the heart was spherical dilatation during systolic period, and the myocardial systolic activities disappeared or significantly weakened	3 m−5 y, some patients were followed up for 3–1 years without recurrence, and 1 patient was followed up for nearly 5 years without recurrence
Li et al. (19)	13	F (38.5%)	50.9 ± 15.8	Subarachnoid hemorrhage	Chest pain, fainting, difficulty breathing	ST-SE and T-WI	The movement of the left ventricular wall was weakened or disappeared, and the apex of the heart was dilated like a ball	No corresponding vascular infarction was observed, and mild coronary stenosis may be present in elderly patients	N

*F, female; ECG, electrocardiogram; UCG, echocardiography; LVG, left ventricular angiography*.

* *ES, emotional stress;*

# *PS, physical stress;*

a *ST-SE, ST-segment elevation;*

b *T-S, ST segment depression;*

c *T-WI, T—wave inversion;*

d *QTc-IP, QTc interval prolongation*.

## Results

### Basic Information

Among the 9 patients with a definite diagnosis of Takotsubo syndrome, 3 cases (33.3%) were male, and 6 cases (66.7%) were female. In this study, the mean age of onset was 46.4 years old, the median age was 42 years old, 6 patients (66.7%) were ≤42 years old, 3 patients (33.3%) were 42 years old, and 2 patients (22.2%) were elderly (≥60 years). The median time from onset to treatment was 1 day, the shortest time was 4 h, and the longest time was 5 years. All patients were treated in the emergency department. There were 2 cases of hypertension, 1 case of diabetes, 1 case of abnormal glucose tolerance, and no patients with related medical history. There were 5 cases in spring, 1 case in summer and 1 case in autumn, and 2 cases in winter. Two patients lived in rural areas, and seven lived in urban areas. None of the above patients had a genetic history of related diseases.

### Clinical Manifestations of Initial Diagnosis

All patients came to the hospital on emergency. The main symptom was chest pain for 8 patients (88.9%) who came to the hospital, and 1 patient went to the hospital because of syncope. Among them, 7 cases (77.8%) were mainly caused by emotional fluctuations or mental stimulation. Among the remaining 2 cases, 1 case developed chest pain without a known cause, and the other case was admitted to the hospital for syncope without a known cause. Seven cases (77.8%) had pain mainly in the precardiac area, one case had pain in the shoulder area, and one case was admitted to the hospital due to syncope, the cause of which was not known. In patients with chest pain symptoms, the nature of chest pain was compression, needle or contraction like, with a median duration of 1 h. The longest duration of symptoms was that the pain was never relieved, and the shortest duration of pain was 3–5 min. Among the alleviating and aggravating factors, 2 cases (22.2%) reported that their symptoms were relieved after oral administration of suxiaojiuxin pills or nitroglycerin and other vasodilators, and 2 cases reported that their symptoms were relieved after rest. Among the accompanying symptoms, 2 patients developed shortness of breath, 3 patients developed nausea and vomiting, 3 patients developed dizziness and headache, and 1 patient developed shock. In the course of diagnosis and treatment, 6 patients visited our emergency department for the first time because of the disease, and the remaining 3 patients were transferred to our emergency department after visiting a local hospital. Regarding surgical history of the patients, 7 cases (77.8%) had no previous operations, 1 case received surgical treatment for a hepatic hydatid, and 1 case received surgical treatment for gallstones. One patient had a history of a tuberculosis infection and has now been cured. Among the 9 patients, 3 patients (33.3%) smoked, including 2 males and 1 female. A total of 2 people drank alcohol, all of them were male, and all of them drank alcohol occasionally. Among the vital signs, 9 patients had normal body temperatures and respiratory rates, 2 had rapid heart rates, 1 had shock, 7 were overweight with a high body mass index, 1 was a woman with a low body weight, and 1 was a woman with normal weight, as shown in Table 2.

**Table 2:** Comparative analysis of clinical characteristics of 9 patients.

**Patient**	**Sex**	**Age**	**Symptoms**	**Onset time**	**Duration**	**Inducement**	**Location**	**Characteristic**	**Aggravating or mitigating factors**	**Accompanying symptoms**	**Follow-up**
1	M	38	Syncope	4 h	None	None	None	None	None	None	50 months, LVEF recovered from 41.3 to 62%, no recurrence occurred
2	F	42	Chest pain	3 days	2 h	Emotional stress	Precordial area	Squeezed	Symptoms relieved after taking 2 capsules of suxiaojiuxin pills	Palpitation, nausea, vomiting	47 months, the admission LVEF was 63%, no recurrence occurred
3	F	42	Chest pain	1 day	10 min	Emotional stress	Precordial area	Needle stick	Can be relieved after rest	Dizziness, tinnitus	33 months, the admission LVEF was 67% and follow-up LVEF was 65%, no recurrence occurred
4	F	60	Chest pain	4 h	1 h	Emotional stress	Behind the sternum	Squeezed	No relief after rest	Flustered	21 months, the admission LVEF was 62.4%, no recurrence occurred
5	F	37	Chest pain	10 days	A few hours	Emotional stress	Precordial area	Compressed, stuffy	Increased after upper respiratory tract infection	Dizziness, vomiting, shock	17 months, the admission LVEF was 66%, no recurrence occurred
6	M	36	Chest tightness	1 day	1 h	Physical stress	Precordial area	Tight	None	Headache, nausea, back discomfort	16 months, the admission LVEF was 60%, no recurrence occurred
7	M	34	Shoulder pain	1day	1 h	Emotional stress	Shoulders, neck, and behind the breastbone	Relief after activity	Relief after activity	None	14 months, the admission LVEF was 63% and follow-up LVEF was 63%, no recurrence occurred.
8	F	50	Chest pain	9 days	0.5 h	None	Middle and posterior sternum	Compressed	Relief after oral suxiao jiuxin pill	Shortness of breath, numbness and weakness in hands	9 months, the admission LVEF was 65%, no recurrence occurred
9	F	79	Chest discomfort	8 h	Not relieved	Emotional stress	Precordial area	Relief after rest	Relief after rest	Suffocated	9 months, the admission LVEF was 58.96%, no recurrence occurred

### Hematological Examination

In all 9 patients, the complete hematological examination included routine blood, biochemical, myocardial enzymes, troponin levels, myoglobin levels, BNP levels, inflammatory indexes and other routine indexes, tests for thyroid function. Testing for a partial improvement of thyroid function, testing for eight types of viruses, including coxsackie virus, Epstein-Barr virus and other indexes. In the routine blood examinations, the white blood cell counts of 7 patients were at normal levels, 2 patients had increased levels, 5 patients had an increased percentages of neutrophils, and 3 patients had an increased percentage of monocytes. On the electrolyte test, 4 cases had hypokalemia and 6 cases had hyponatremia. One case had acute renal insufficiency, 1 case had acute left heart failure, 1 case had high triglycerides, and 1 case had a level of low density lipoprotein on the high end of normal. There were 5 cases with creatine kinase elevations, 3 cases with creatine kinase isoenzymes, 2 cases of high troponin levels, 3 cases with different levels of interleukin −6 and the rise of hypersensitive c-reactive protein, and 1 case with subclinical hypothyroidism. Virus 8, Coxsackie virus, and Epstein-Barr virus tests were negative. A paired *T*-test was performed on the routine blood tests before and after admission, and only the counts of the white blood cells and the neutrophils were statistically significant in the comparison (see Tables 3, 4).

**Table 3:** Comparative analysis of blood routine of 7 patients before and after admission.

	**Admitted**	**Discharged**	***t***值	**95%CI**	***P***
WBC	10.59 ± 4.94	8.02 ± 3.37	3.2	0.61–4.54	0.02
RBC	4.68 ± 0.62	4.66 ± 0.55	0.214	0.24–0.29	0.84
Hemoglobin	135.57 ± 16.49	134.86 ± 15.23	0.272	−5.71–7.14	0.79
Platelet	253.57 ± 95.83	232.00 ± 101.39	2.337	−1.00–43.58	0.06
The percentage of neutrophils	78.39 ± 11.27	69.21 ± 14.12	1.598	−4.87–23.21	0.16
Percentage of lymphocytes	14.71 ± 10.49	21.24 ± 8.19	−1.745	−15.68–2.62	0.13
Percentage of monocytes	6.41 ± 3.25	8.14 ± 6.42	−0.852	−6.69–3.23	0.43
Percentage of eosinophils	0.27 ± 0.36	1.04 ± 1.07	−1.861	−1.79–0.24	0.11
Percentage of basophils	0.21 ± 0.12	0.36 ± 0.19	−1.594	−0.36–0.08	0.16
Neutrophils count	8.54 ± 4.58	5.81 ± 3.39	2.664	0.22-5.23	0.04
Lymphocyte count	1.32 ± 0.65	1.54 ± 0.49	−0.926	−0.79–0.36	0.39
Monocyte count	0.69 ± 0.59	0.57 ± 0.35	0.578	0.42–0.68	0.58
Eosinophil count	0.02 ± 0.02	0.06 ± 0.05	−1.875	−0.11–0.01	0.11
Basophil count	0.02 ± 0.01	0.03 ± 0.01	−0.934	−0.02–0.01	0.39
Hematocrit	41.01 ± 5.36	40.73 ± 3.99	0.341	−1.76–2.34	0.75
Mean erythrocyte volume	87.71 ± 3.37	87.66 ± 3.46	0.069	−1.97–2.08	0.95
Mean hemoglobin	29.03 ± 1.67	28.98 ± 1.53	0.183	−0.53–0.62	0.86
Average hemoglobin concentration	331.00 ± 9.98	330.86 ± 10.02	0.049	−6.97–7.26	0.96
Red blood cell distribution width	12.81 ± 0.64	12.93 ± 0.79	−0.745	−0.49–0.26	0.48
Mean platelet volume	10.93 ± 1.18	11.06 ± 1.35	−0.721	−0.56–0.31	0.5
Thrombocytopenia	0.27 ± 0.08	4.06 ± 10.12	−0.992	−13.15–5.56	0.36
Width of platelet distribution	13.16 ± 2.64	13.38 ± 3.45	−0.357	−1.77–1.32	0.73

*P < 0.05 was considered statistically significant*.

**Table 4:** Comparative analysis of biochemical indexes of 5 patients before and after admission.

	**Admitted**	**Discharged**	***t***值	**95%CI**	***P***
K	4.07 ± 0.78	3.88 ± 0.27	0.51	−0.82–1.19	0.64
Na	142.56 ± 13.82	137.04 ± 3.85	0.90	−11.48–22.53	0.42
CL	104.20 ± 2.83	106.20 ± 4.04	−1.59	−5.49–1.49	0.19
Ga	2.21 ± 0.18	2.26 ± 0.12	−0.59	−0.29–0.19	0.59
Uric acid	4.02 ± 2.17	3.83 ± 1.42	0.30	−2.74–3.31	0.78
Creatinine	68.77 ± 46.39	46.61 ± 9.78	0.95	−57.92–106.88	0.41
Total bilirubin	14.49 ± 5.56	14.01 ± 7.67	0.21	−5.79–6.76	0.84
Aspartate aminotransferase	40.67 ± 32.19	55.15 ± 39.73	−0.69	−73.12–44.16	0.53
Lactate dehydrogenase	177.64 ± 72.56	161.35 ± 27.73	0.74	−74.26–118.94	0.52
Creatine kinase	276.06 ± 376.22	120.99 ± 118.84	0.90	−323.02–633.17	0.42
Creatine kinase isoenzyme	17.49 ± 13.63	12.84 ± 8.34	0.72	−13.24–22.53	0.51
Troponin	0.03 ± 0.04	0.01 ± 0.02	1.23	−0.05–0.08	0.34
NT−BNP^*^	53.81 ± 46.74	19.24 ± 17.39	0.64	−708.74–783.98	0.64
Calcitonin original	2.54 ± 5.00	0	1	−0.18–0.21	0.5
Interleukin−6	237.40 ± 442.09	3.74 ± 0.01	0.47	−41.9–45.3	0.72
CRP^#^	15.81 ± 23.39	0.62 ± 0.24	2.24	−1.09–1.57	0.27

* *BNP, N-terminal B-type natriuretic peptide precursor;*

# *CRP, Hypersensitive C-reactive protein; P < 0.05 was considered statistically significant*.

### Imaging Examination

**Figure 2:**
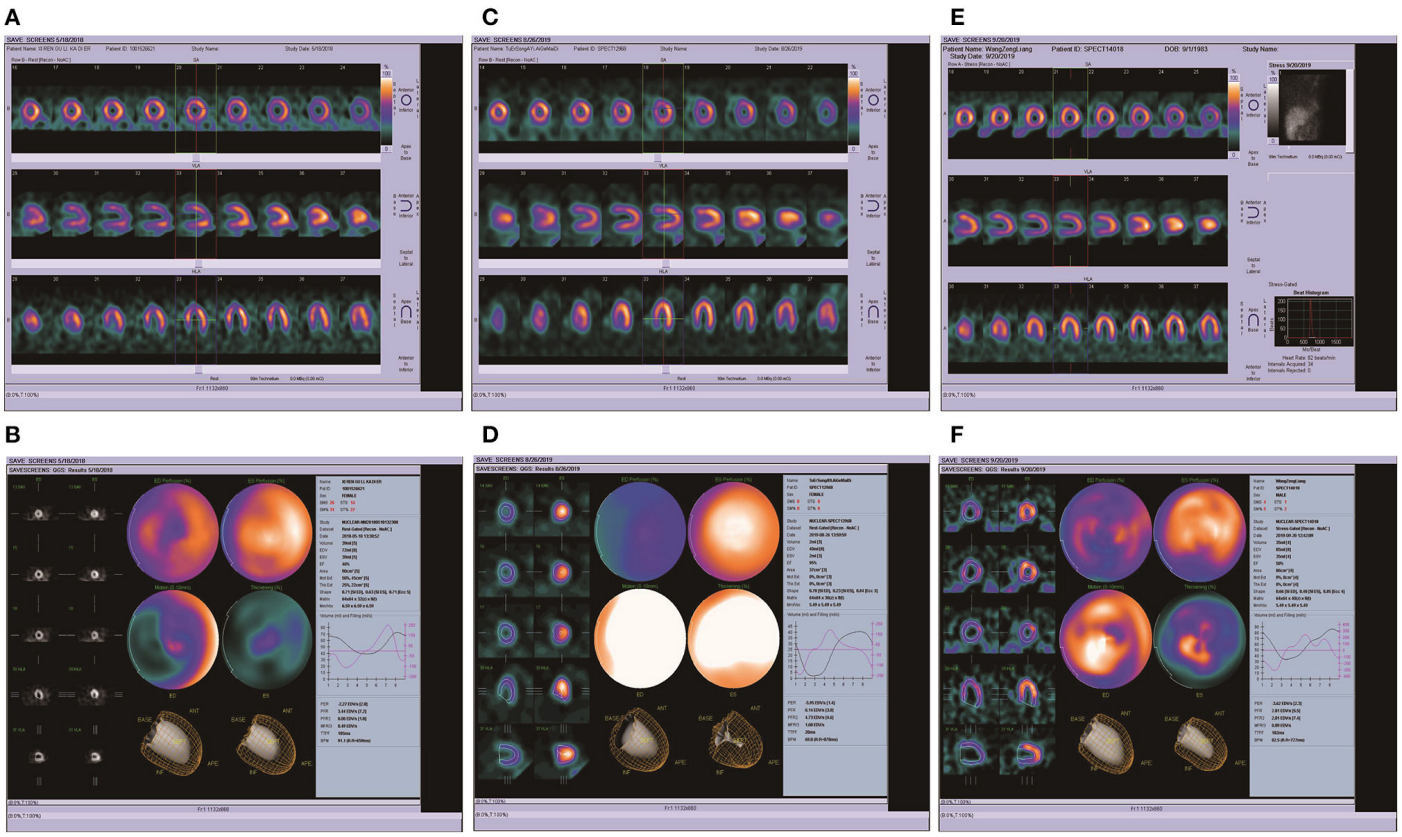
ECT imaging results of 3 patients. **(A,B)** The ECT of patient 3, the results of myocardial ECT showed that EF 46%, PFR 3.44, decreased myocardial perfusion in the left ventricular apex, significantly decreased left ventricular septal wall motion, slightly decreased apical and anterior wall motion, decreased left ventricular systolic function, and normal diastolic function. **(C,D)** The ECT of patient 5, the results of myocardial ECT showed that EF 95%, PFR 0.14, the left ventricle heart cavity is small, no blood loss in each wall. **(E,F)** The ECT of patient 6, the results of myocardial ECT showed that EF 58%, PFR 2.81, slight absence of blood samples in anterior wall and lateral wall of left ventricle.

Among the imaging examinations conducted during the hospitalization of the patients, 3 patients had a complete myocardial radionuclide examination, 1 patient had a decreased left ventricular wall motion, and 1 patient had a decreased LVEF. Case 1 was hospitalized due to syncope, and no obvious abnormalities were found in pulmonary artery CTA, brain NMR and brain enhanced NMR, as shown in Figure 2.

### Coronary Angiography

**Figure 3:**
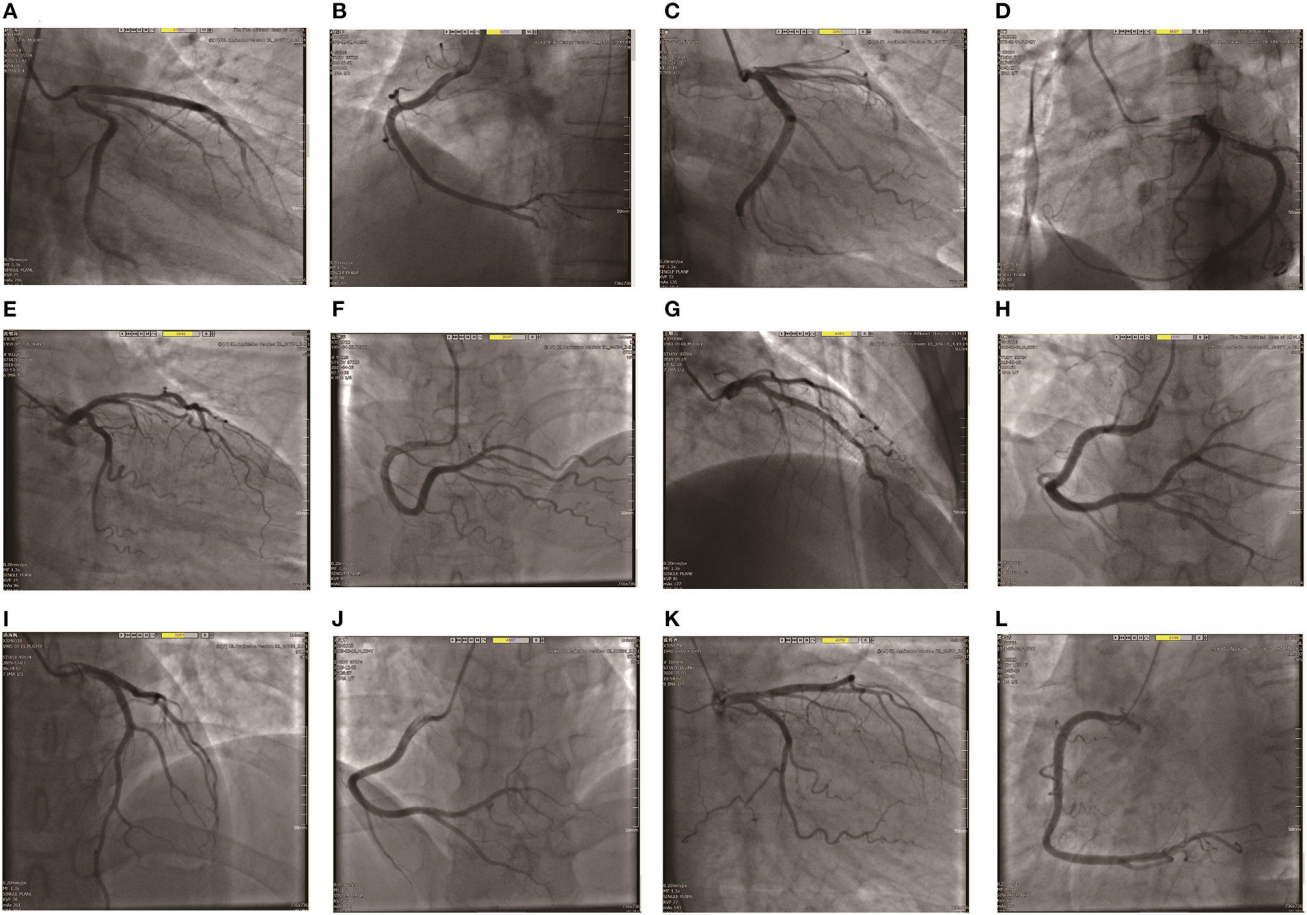
Coronary angiographic images of 6 patients. **(A,B)** The Coronary angiography of patient 1, which suggested slow coronary blood flow, LAD TIMI1-2; LCX TIMI1-2; Middle branch TIMI 2; RCA TIMI. **(C,D)** The Coronary angiography of patient 2, LAD30% localized TIMI2, the rest TIMI3; **(E,F)** The Coronary angiography of patient 4, LADD1 proximal 60%, LAD middle 40% TIMI3. **(G,H)** The Coronary angiography of patient 6, Coronary angiography showed no significant stenosis. **(I,J)** The Coronary angiography of patient 7, Coronary angiography showed no significant stenosis. **(K,L)** The Coronary angiography of patient 9, The proximal LAD was limited to 40% stenosis, the middle RCA was limited to 30%, and the blood flow was slow throughout the LCX.

All 9 patients underwent emergency coronary angiography. Case 1 had slow coronary blood flow; and 30% of the patients with LAD had proximal stenosis. In case 4, 40% of the coronary LAD was narrowed. In case 9, the proximal LAD had a localized narrowing of 40%, the middle RCA was narrowed by 30%, and the whole LCX had a slow blood flow, as shown in Figure 3.

### ECG

**Figure 4:**
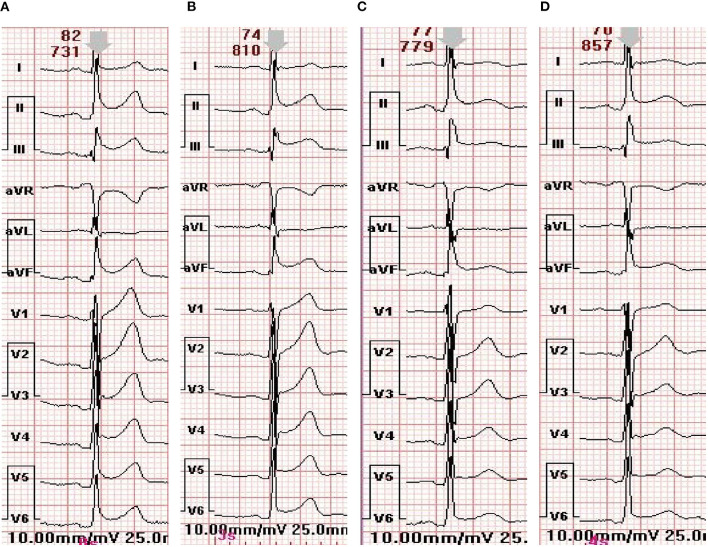
ECG changes of patient 7 during hospitalization. **(A)** The electrocardiogram at admission. **(B)** The electrocardiogram on the first day of admission. **(C)** Electrocardiogram on the third day of admission. **(D)** The electrocardiogram before discharge.

**Figure 5:**
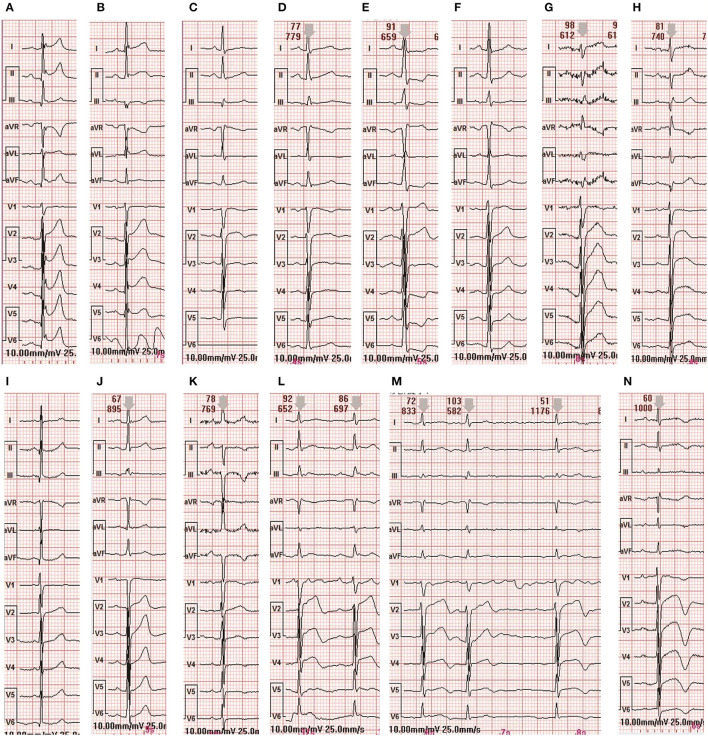
ECG changes in 8 patients during hospitalization **(A,B)** are the ECG of admission and discharge of case 1 **(C,D)** are the ECG of admission and discharge of case 2 **(E,F)** are the ECG of admission and discharge of case 3 **(G,H)** are the ECG of admission and discharge of case 4 **(I)** is the ECG of admission and discharge of case 5 **(J)** is the ECG of admission and discharge of case 6 **(K,L)** are the ECG of admission and discharge of case 8 **(M–O)** are the ECG of admission and discharge of case 9.

In the ECG examination, a paired *T*-test of objective ECG indexes from admission to discharge was conducted for 9 patients. The P wave time limit, QT, QTC, QRS time limit and P-R interval between admission and discharge were not statistically significant (*P* > 0.05). After characteristic analysis of the 9 cases of patients, we found that in cases 1 and 7 the ST was prolonged, in case 5, the ST segment was decreased and in cases 4, 8, 2, and 9, there was an ST -T change. There were 9 cases of paroxysmal atrial flutter and paroxysmal atrial fibrillation. Patients 2, 3, 4, and 9 showed prolonged QTc interval in ECG examination, while the rest of the patients were normal. Objective indicators of ECG are shown in Table 5. ECG images are shown in Figures 4, 5.

**Table 5:** Comparison of P, QT, QTC, QRS, and P-R intervals in ECG of 6 patients before and after admission.

	**Admitted**	**Discharged**	***t***值	**95%CI**	***P***
P (ms)	98.67 ± 7.00	98.67 ± 11.98	0	−18.54–18.54	1
QT (ms)	334.33 ± 95.55	401.33 ± 63.97	−1.06	−229.99–95.99	0.34
QTc (ms)	402.67 ± 92.31	445.33 ± 49.90	−0.81	−178.34–93.01	0.46
QRS (ms)	81.83 ± 19.76	86.33 ± 5.72	−0.49	−28.08–19.08	0.64
P-R (ms)	160.00 ± 22.31	172.67 ± 23.79	−1.77	−31.09–5.76	0.14

*P < 0.05 was considered statistically significant*.

### Ultrasound Examination of Cardiac and Cervical Vessels

**Figure 6:**
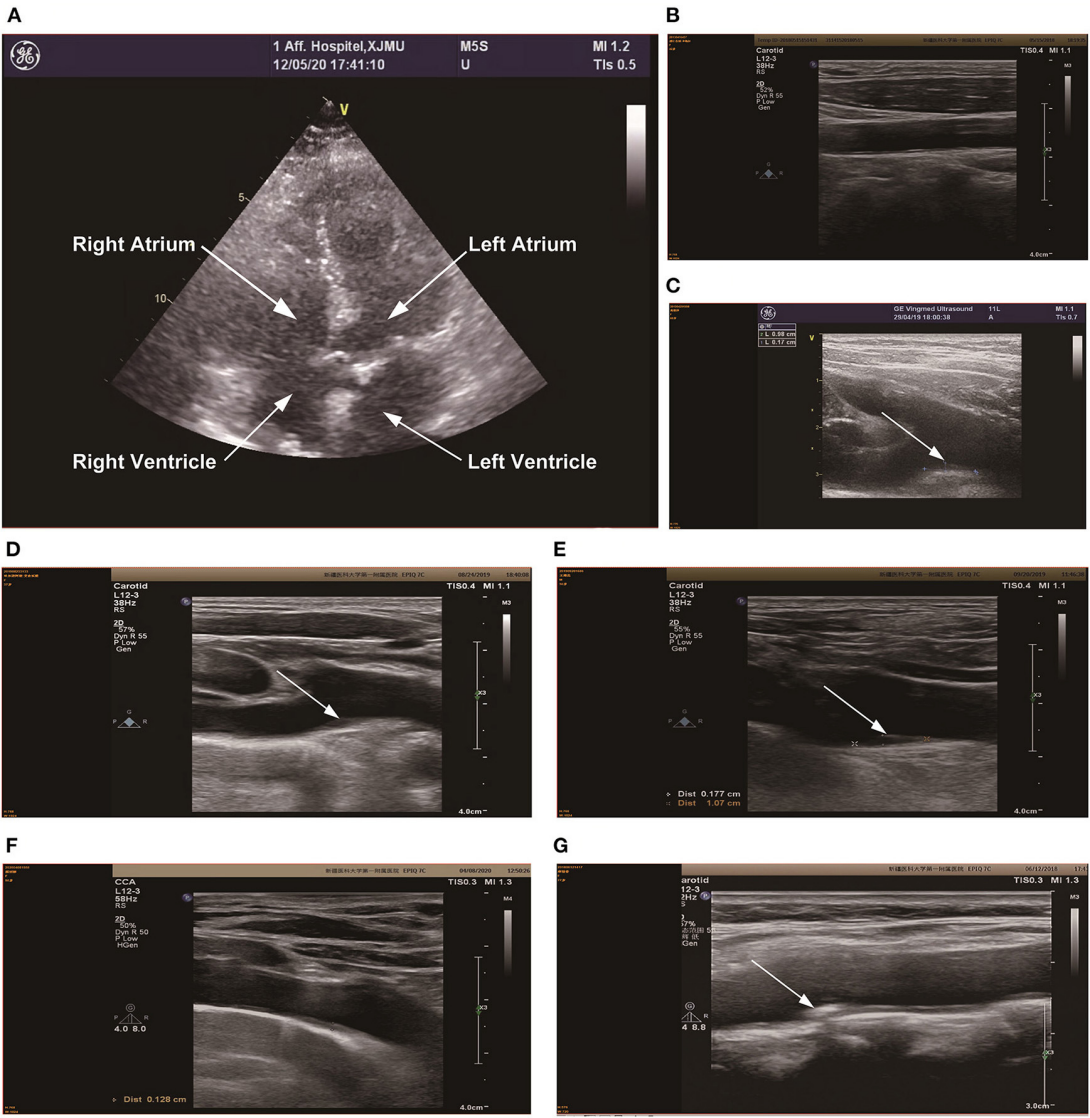
Color Doppler ultrasonography of the neck and heart was performed in 6 patients. **(A)** The heart color Doppler ultrasound image of patient 9, and the results suggested left atrial enlargement, reduced left ventricular myocardial phase motion, mitral valve insufficiency, active surface insufficiency, and mild pulmonary hypertension. **(B)** is the cervical vascular color Doppler ultrasound of patient 3, there were no obvious abnormalities in cervical vascular ultrasound. **(C)** is the cervical vascular color Doppler ultrasound of patient 4, and the results suggested that the innameless artery bifurcation and the plaque size of the right subclavian artery was about 9.8 × 1.7 mm, which has been marked in the figure. **(D)** is the cervical vascular color Doppler ultrasound of patient 5, and the results suggested that the innameless artery bifurcation and the plaque size of the right subclavian artery was about 10 × 1.8 mm, which has been marked in the figure. **(E)** is the cervical vascular color Doppler ultrasound of patient 6, and the results suggested that the innameless artery bifurcation and the plaque size of the right subclavian artery was about 11.1 × 1.8 mm, which has been marked in the figure. **(F)** is the cervical vascular color Doppler ultrasound of patient 8, intima thickening of the left carotid artery (1.2 mm), bifurcation of the innominate artery and thickening of the right subclavian artery (1.3 mm), which has been marked in the figure **(G)** is the cervical vascular color Doppler ultrasound of patient 9, there were no obvious abnormalities in cervical vascular ultrasound.

During the cardiac color ultrasound examination, the LVEF of Case 1 was significantly impaired with a weakened ventricular wall motion, but the ejection fraction returned to normal upon discharge. Aortic insufficiency was observed in case 4. Minor aortic regurgitation occurred in case 7. In case 9, there was left atrium enlargement, reduced left ventricular muscle segmental motion, mitral valve insufficiency, aortic valve insufficiency, and mild pulmonary hypertension. Other patients showed different degrees of ventricular wall motion reduction. The ventricular wall movement gradually returned to normal during out-of-hospital follow-up. See Figure 6.

In the 9 patients, we found that, except for patients 1, 2, and 7 who did not receive cervical artery ultrasound, all the other patients had plaques located in the bifurcation of the innaminate artery and the right subclavicular artery to varying degrees, and the maximum plaques were ~11.1 × 1.8 mm. See Figure 6, Color ultrasound of remaining neck vessels is shown in Figure 6.

## Treatment and Follow-Up

For patients admitted to hospital for treatment of chest pain, the rise of the dynamic changes, the electrocardiogram (ECG) changes and rise in heart markers, the emergency department considered the possibility of coronary heart disease (CHD). Hence, the emergency departments considered performing emergency coronary angiography examinations, hospitalization and nutrition to improve myocardial blood flow, reduce myocardial oxygen consumption, and secondarily prevent coronary heart disease, and they performed other comprehensive treatments, The patient's symptoms were significantly improved at discharge from the hospital compared with those on admission. Of course, Takotsubo syndrome may also cause serious complications, such as heart failure, malignant arrhythmia, hemodynamics and other complications. Case 1 developed shock combined with acute renal failure, and Case 9 developed acute left heart failure accompanied by paroxytic atrial fibrillation, which improved after symptomatic treatment and was discharged. None of the 9 patients had any related complications or recurrence during the out-of-hospital follow-up. After discharge, due to the poor compliance of some patients, the patients stopped taking the medications, while some patients underwent medication adjustments under the guidance of doctors.

## Discussion

TTS was first described in 1990. Japanese scholar Dote et al. (20) reported the left ventricular angiographic images of 5 patients without obstructive coronary artery disease but with symptoms of myocardial infarction and defined it as TTS. The clinical symptoms and electrocardiogram manifestations of this syndrome are similar to those of acute coronary syndrome. The main characteristic of this syndrome is regional abnormal left ventricular wall motion accompanied by abnormal left ventricular dilation during the systolic period. After the relevant inducement was removed, the cardiac function of the patient returned to normal, and the prognosis was generally good.

As research on TTS continues, an epidemiological study by Murugiah et al. (21) found that the incidence of TTS increased from 2.3 cases per 100,000 population years in 2007 to 7.1 cases per 100,000 population years in 2012. A study of 1,750 TTS by Templin et al. (22) found that 89.8% were female, with an average age of 67 years. TTS has been found to occur in children in some published case reports (22–24). Among the TTS patients in this study, women accounted for 66.7% of cases. A total of 264 cases of TTS were seen in the Chinese population, among which women accounted for 78.8% of cases. The main reason may be that estrogen regulates sympathetic tension through the central action of the myocardium, vascular system and βAR expression and contributes to the relative inhibition of βAR expression during the female reproductive period. This sympathetic lytic effect of estrogen disappears after menopause, and the myocardial and vascular responses induced by βAR agonists are enhanced. This may partly explain why TTS is most common in postmenopausal women (25).

The main causes of TTS are emotional stress or physical stress, and it has been found that pheochromocytoma, subarachnoid hemorrhage and other diseases can cause TTS. Sharkey et al. (23) followed up 136 TTS patients and found that 22 cases were related to physical stress, 15 of which had no obvious stress inducement. Wittstein et al. (26) found that catecholamine levels were higher in 19 TTS patients than in 7 Killipi II AMI patients. We think that emotional or physical stress activates the adrenal medulla in the human body, leading to an increased secretion of catecholamines in the human body, and increased catecholamine reduces the activity of cardiomyocytes through periodic calcium overload mediated by AMP 8, thus leading to a series of syndromes caused by a high catecholamine in patients.

Furthermore, we found that the main clinical symptoms of TTS patients were chest pain, including chest tightness, palpitations, dyspnea, syncope, fatigue and other symptoms. One reason for the symptoms of chest pain in patients includes the spasm of multiple coronary arteries. Dote et al. (20) believed that left ventricular dysfunction and myocardial shock are caused by spasms of multiple coronary arteries at the same time. A second reason could be due to coronary microvascular dysfunction. A study by Fazio et al. (27) found slow coronary blood flow in 23 patients by evaluating TIMI frame count in 24 TTS patients. Finally, transient thrombosis in the anterior descending branch caused myocardial infarction. Ibanez et al. (28) conducted intravascular ultrasound examinations on 5 TTS patients and found that atherosclerotic plaque rupture in the anterior descending branch resulted in thrombosis. However, among the TTS patients we collected, there were 2 cases with slow coronary blood flow, and none of the patients showed obvious signs of coronary artery stenosis, which was consistent with the manifestations of TTS. The specific pathogenesis of TTS still needs further study.

After admission, we performed serological and related auxiliary examinations on TTS patients, and paired *T*-tests were performed on routine blood, biochemical, myocardial markers, and inflammatory indicators before and after admission. Only the counts of white blood cells and neutrophils were statistically significant. Morel et al. (29) studied 17 patients with TTS and found that the CRP of TTS patients was significantly elevated at admission, which was correlated with the LVEF and BNP. An endocardial biopsy of TTS patients found that the reversible infiltration of inflammatory cells may be related to sympathetic nerve excitation, and cytokines and reactive oxygen species released by activated inflammatory cells may lead to myocardial injury, thereby causing an increase in inflammatory cells and myocardial markers. Furthermore, the improvement of heart color Doppler ultrasound examination revealed that TTS patients had different degrees of reduced left ventricular ejection fraction and abnormal ventricular wall motion, and typical patients could also have spherical apex dilatation. After follow-up, most patients had recovered their LVEF and ventricular wall motion. In a case-control study of 1,750 patients with TTS and ACS, it was found that TTS had a significantly lower left ventricular ejection fraction (22). Citro et al. (30) found that patients in the LVEF ≤35 group were older than those in the LVEF >35 group and were more likely to have adverse cardiovascular events. Abumayyaleh et al. (31) found that after 5 years of follow-up, the long-term mortality of TTS patients was significantly higher than that of ACS patients. In a study of abnormal left ventricular wall motion and coronary artery flow, it was found that during the acute phase of TTS, the myocardial blood flow of the dysfunctional left ventricular segment was lower than that of the normal ventricular wall motion (32, 33). In summary, the study found that the main manifestation of TTS cardiac ultrasound is a disease with a good prognosis and a reduced LVEF. However, if the patient is an elderly female with multiple underlying diseases with an ejection fraction of ≤35%, the patient likely has a poor prognosis. In addition, we also found plaque formation in the bifurcation of the innamental artery and the right subclavian artery in 5 patients with complete cervical artery color Doppler ultrasound. Currently, there is no relevant study on carotid artery plaque and TTS, and the mechanism is not yet clear.

In addition, the ECG of TTS patients showed the same ECG manifestations as ACS, such as ST segment elevation, ST segment depression and T wave inversion, accompanied by varying degrees of QT interval prolongation. Some studies believe that the prolonged QT interval may be due to the abnormal expansion of the left ventricular myocardium caused by the increased secretion of catecholamine and the temporary abnormal repolarization and dispersion caused by myocardial edema at the apex and base of the left ventricle. However, after 105 ± 32 days of TTS follow-up by Kurisu et al. (34), all ECG indicators of the patient basically returned to normal. An observational study of TTS by Madias et al. (35) found that corrected QT interval prolongation was associated with the occurrence of ventricular arrhythmias. Although ECG is normal in a few TTS patients, most TTS patients present with ACS-like ECG abnormalities on admission. Frangieh et al. (36) developed a standard to distinguish TTS from AMI, regardless of the presence of ST-elevation or non-ST-elevation myocardial infarction. Patients with TTS showed related ECG changes, and after follow-up, the ECG of all TTS patients returned to normal.

Three patients with TTS were hospitalized for single photon emission computed tomography scans (SPECT), and the results showed that 1 case with left ventricular wall motion was abated, and was accompanied by a significantly lower ejection fraction. There were different degrees of blood flow deficiencies in the left ventricle and apex. Some foreign studies have found that TTS patients may have abnormal perfusion or low and normal coronary blood flow reserve (37–39). Yoshida and Camici's study (40, 41) found that TTS reversibly decreased coronary blood flow reserve, but this was not associated with vascular structure changes. Combined with domestic and foreign studies, we found that TTS patients may have abnormal coronary blood perfusion, which may be caused by vascular spasm causing abnormal coronary blood perfusion, resulting in patients with various chest pain, breathing difficulties and other symptoms.

TTS is a group of clinical syndromes induced by mental or physical stimulation, with chest pain as the main symptom, partly accompanied by an increase in white blood cells, neutrophils, creatine kinase, troponin, prolonged QTC interval, and no obvious coronary stenosis. The prognosis is generally good, with few serious complications. In clinical practice, since the symptoms of this disease are similar to those of acute coronary syndrome, careful attention should be given to improving the diagnostic efficiency of TTS to reduce the symptoms.

### Limitations

The limitations of this study were that the sample size was small, the average follow-up time was short, and typical left ventricular angiography images of patients with Takotsubo syndrome were not obtained due to posture or technical problems at that time, or because the possibility of this disease was not considered at that time. The next step of our research plan is to strengthen the research on the pathophysiological and other potential mechanisms of Takotsubo syndrome as well as the research on hematological biomarkers, and focuses on establishing a multi-center, large-sample, long-follow-up case study bank.

## Data Availability Statement

The raw data supporting the conclusions of this article will be made available by the authors, without undue reservation.

## Ethics Statement

Ethical review and approval was not required for the study on human participants in accordance with the local legislation and institutional requirements. The patients/participants provided their written informed consent to participate in this study. Written informed consent was obtained from the individual(s) for the publication of any potentially identifiable images or data included in this article.

## Author Contributions

YM and XX supervised the project and designed the study. JY was responsible for analyzing and interpreting the data and wrote the manuscript. CD and QY were responsible for collecting, collating, and statistical data. SC and MM participated in part of revising work. All authors have read and approved the manuscript.

## Funding

This research was funded by the National Natural Science Foundation of China (91957208, 81770235, and 81960046), State Key Laboratory of Pathogenesis, Prevention and Treatment of High Incidence Diseases in Central Asia Fund (SKL-HIDCA-2019-4), and Prevention and control of major chronic Non-communicable disease Project (2018YFC1311505).

## Conflict of Interest

The authors declare that the research was conducted in the absence of any commercial or financial relationships that could be construed as a potential conflict of interest.

## Publisher's Note

All claims expressed in this article are solely those of the authors and do not necessarily represent those of their affiliated organizations, or those of the publisher, the editors and the reviewers. Any product that may be evaluated in this article, or claim that may be made by its manufacturer, is not guaranteed or endorsed by the publisher.
